# Bioactive Compounds from Seaweeds as Natural Preservatives for Seafood

**DOI:** 10.3390/molecules31183317

**Published:** 2026-09-18

**Authors:** Janine Ametin, Anil Kumar Anal, Muspirah Djalal, Yun-Hsin Lin, Hui-Wen Lin, Kuan-Chen Cheng

**Affiliations:** 1 Faculty of Food, Agriculture and Natural Resources, Asian Institute of Technology, Klong Luang, Bangkok 12120, Thailand; janine.ametin@nfrdi.da.gov.ph (J.A.); anilkumar@ait.ac.th (A.K.A.); 2 Fisheries Postharvest Research and Development Division, National Fisheries Research and Development Institute, Quezon City 1103, Philippines; 3 Institute of Food Science and Technology, College of Bioresources and Agriculture, National Taiwan University, Taipei 10617, Taiwan; 4 Institute of Biotechnology, National Taiwan University, Taipei 10617, Taiwan; 5 Institute of Medicine, Chung Shan Medical University, Taichung 402, Taiwan; 6 Department of Medical Research, Chung Shan Medical University Hospital, Taichung 402, Taiwan; 7 Department of Optometry, Asia University, Taichung 413305, Taiwan; 8 Department of Medical Research, China Medical University Hospital, Taichung 404327, Taiwan

**Keywords:** seaweed bioactives, packaging, seafood preservation

## Abstract

Escalating environmental concerns over petroleum-based plastics have driven growing interest in seaweed-derived polysaccharides and bioactive compounds as sustainable alternatives for biodegradable packaging. This review synthesizes recent advances in seaweed-based packaging, particularly related to seafood preservation. It covers the development of edible films and coatings, innovations in active and smart packaging, and the creation of multilayer multifunctional systems. The review highlights key applications that demonstrate the effectiveness of seaweed-based materials in delaying microbial spoilage, reducing lipid oxidation, preserving sensory quality, and extending the shelf life of seafood. Additionally, this review addresses food safety and regulatory considerations and provides a detailed examination of major barriers to commercial adoption, particularly variability in composition depending on environmental, physiological, extraction, and geographical factors, high production costs associated with polysaccharide extraction and purification, insufficient pilot- and industrial-scale validation, and the lack of standardized processing protocols.

## 1. Introduction

Packaging acts a critical barrier that protects food from environmental hazards, degradation, and contamination throughout processing, storage, transportation, and distribution. By extending product shelf life, packaging reduces food waste and communicates essential product information to consumers [1]. Petroleum-based packaging is extensively utilized in the food industry because of its structural durability, efficient barrier properties, and low production cost. However, its environmental persistence, role in microplastic pollution, and reliance upon fossil resources have created an urgent demand for renewable, biodegradable, and environmentally friendly alternatives that maintain comparable performance [2].

Marine biomass, particularly seaweed, has gained considerable attention as sustainable feedstock for biodegradable packaging because of its rapid growth, high biomass productivity, and minimal requirements for freshwater, fertilizers, and arable land [3,4]. In addition to these environmental benefits, seaweed contains a diverse range of valuable compounds, such as polysaccharides, proteins, pigments, minerals, polyphenols, lipids, vitamins, and other bioactive metabolites [5]. Polysaccharides exhibit strong gelling and film-forming properties, and along with other bioactive compounds present in seaweed, provide antioxidant and antimicrobial effects that improve food preservation and extend the shelf life of perishable products [6].

Seafood products, such as fish, crustaceans, and mollusks, are among the most valuable food commodities globally. They contribute substantially to food security, human nutrition, and the livelihoods of millions of individuals. These products serve as rich sources of high biological-value protein, omega-3 fatty acids, vitamins, and essential minerals, all of which are associated with significant health benefits [7]. However, due to their high moisture content, close to neutral pH, and nutrient-rich matrices, seafood products are highly perishable and susceptible to microbial and enzymatic degradation after harvest [8,9]. Effective packaging is essential for maintaining product freshness, preserving nutritional quality, minimizing postharvest losses, and extending the marketability of these products during storage and distribution. As sustainability becomes a priority in the global food industry, research on biodegradable materials that enhance seafood safety and protect the environment is increasingly important [10].

Although considerable progress has been made, several challenges continue to impede the commercial adoption of seaweed-based packaging. Key limitations include sub-optimal mechanical properties, material degradation, variability in seaweed biomass, processing scalability, production costs, and regulatory and safety concerns, all of which require further investigation [3,6]. Previous review articles have provided valuable insights into seaweed-based packaging, edible films and coatings, marine polysaccharides, and active food packaging. However, these studies have largely focused on seaweed polysaccharides, specific packaging methods, or broader food applications. In contrast, this review provides an integrated, seafood-focused perspective by linking the chemical composition and techno-functional properties of various seaweed-derived components with their specific packaging functions and roles in seafood preservation and freshness monitoring. The review first examines the principal seaweed-derived polymers and bioactive compounds relevant to packaging, along with the main extraction methods used to obtain functional seaweed components. It then explores the relationship between major seafood spoilage mechanisms and conventional freshness indicators, establishing a foundation for understanding how packaging properties and functionalities can delay deterioration or monitor quality changes. The review then analyzes recent developments in seaweed-based edible films and coatings, active and smart packaging systems, and emerging multilayer, composite, and multifunctional approaches, emphasizing the distinct roles of seaweed polysaccharides, isolated bioactive compounds, and whole seaweed extracts. Particular attention is given to the physicochemical, barrier, mechanical, antioxidant, antimicrobial, moisture-regulating, and pH-responsive properties of these materials and their contributions to maintaining seafood quality, extending shelf life, and enabling freshness monitoring. Finally, the review critically addresses the technological, economic, regulatory, and safety challenges that limit the practical application of seaweed-based packaging and identifies future research directions for developing scalable, sustainable, and high-performance systems for seafood preservation and freshness monitoring.

## 2. Biopolymers and Bioactives in Seaweed

**Figure 1:**
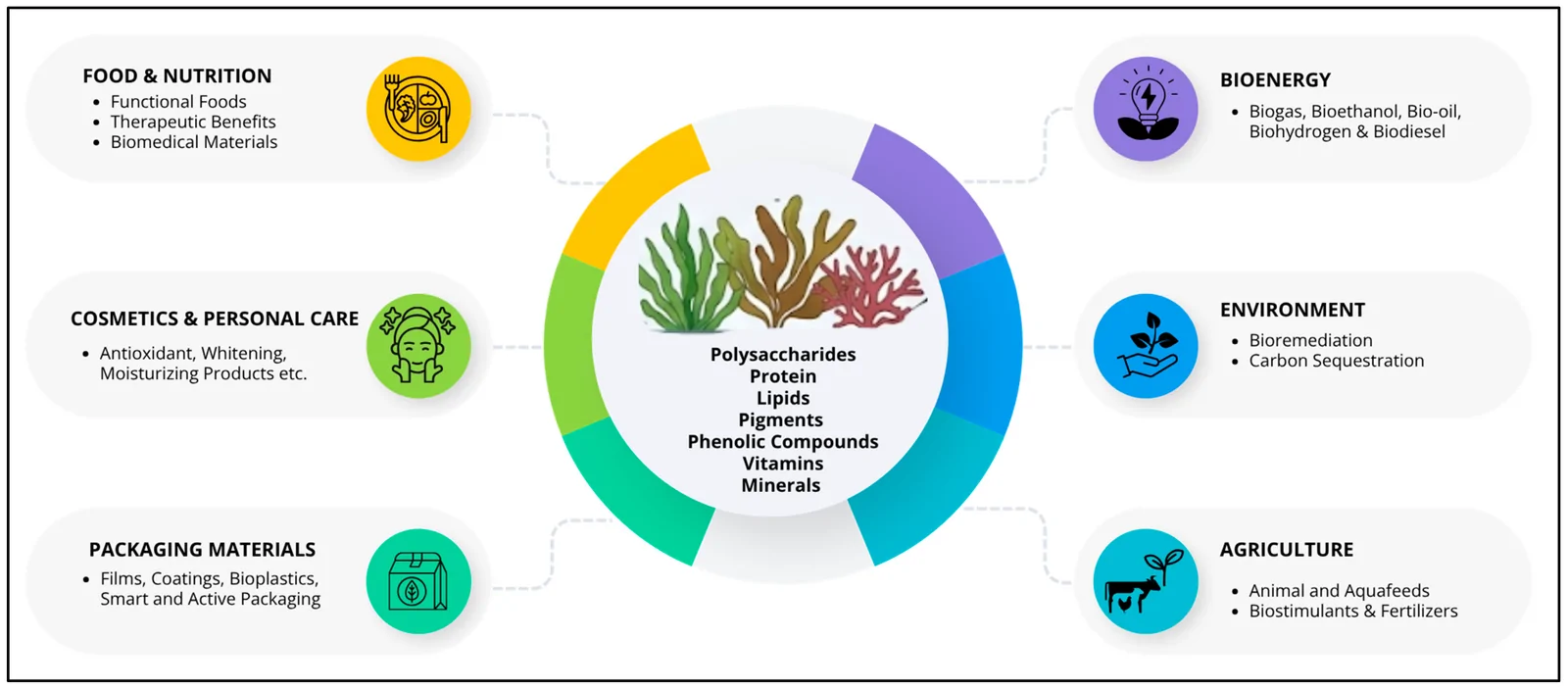
Application of Seaweed Bioactives.

Seaweeds are classified into three rimary taxonomic groups based on pigmentation and species diversity: red (*Rhodophyta*), brown (*Ochrophyta*, *Phaeophyta*), and green (*Chlorophyta*) [11,12]. They are rich in essential macromolecules such as polysaccharides, proteins, and lipids. They also contain many bioactive compounds, including minerals, vitamins, pigments, polyphenols (such as phlorotannins and bromophenols), and carotenoids (e.g., fucoxanthin and astaxanthin). The physicochemical and biological properties of these compounds, as well as their composition, vary according to species, seasonality, cultivation, geographic origin, and extraction methods. These characteristics enable the conversion of seaweeds into a diverse array of high-value products, including applications in food, agriculture, environmental remediation, and biomaterials (Figure 1) [5,13,14].

### 2.1. Seaweed Polysaccharides

**Figure 2:**
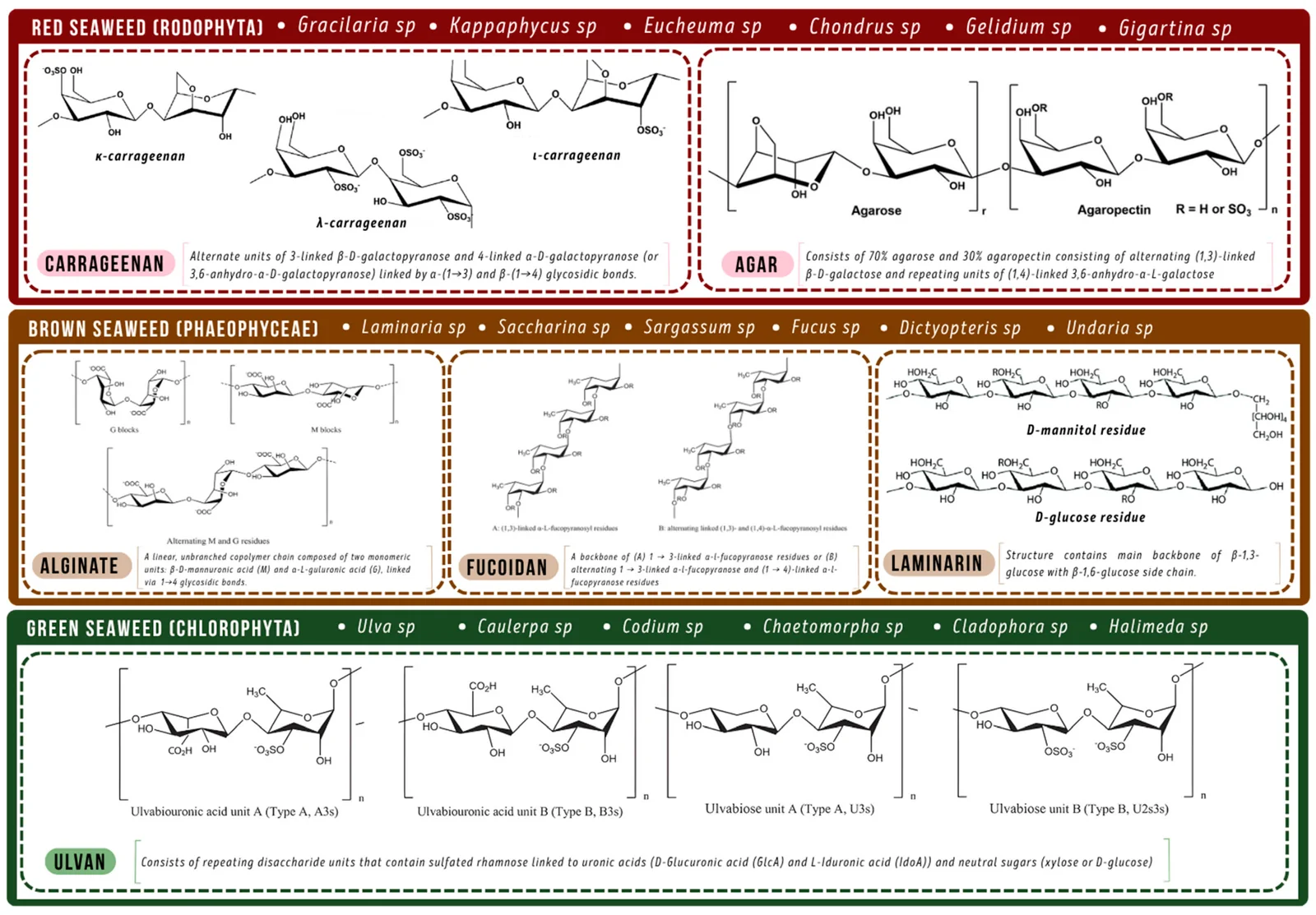
Seaweed Polysaccharides with Packaging Potential (Adapted from [3]).

Polysaccharides constitute the primary component of seaweeds, accounting for 50–74% of their dry weight, and are recognized as versatile biopolymers with a broad spectrum of biological activities [5,11]. Seaweed-based packaging materials are primarily derived from three main polysaccharides: alginate, carrageenan, and agar [13]. Emerging materials such as ulvan, fucoidan, and laminarin from green and brown seaweeds, respectively, also demonstrate potential [15]. Their functionality is determined by their molecular weight, chain conformation, glycosidic linkages, and degree of sulfation (Figure 2) [16,17]. This structural diversity results in functionalities such as gel formation, emulsification, viscosity modulation, water-holding capacity, film formation, and bioactivity. These characteristics offer advantages for packaging applications; however, they result in films that exhibit varying techno-functional properties [3,14].

Alginate, derived mainly from brown seaweeds, including *Saccharina*, *Laminaria*, and *Sargassum* species, consists of (1→4)-linked β-D-mannuronic acid (M) and α-L-guluronic acid (G) units. The relative proportion (M/G ratio) and arrangement of these units determine their rheological behavior, gel strength, and functional performance. Alginates with a lower M/G ratio form stronger, more brittle gels, whereas a higher ratio yields softer, more flexible, and elastic gels. G-blocks that are more rigid can interlock with divalent cations like calcium (Ca^2+^) to form a three-dimensional “egg-box” hydrogel network [15,17,18]. Alginate is among the most extensively utilized seaweed-derived polysaccharides in food packaging, owing to its strong film-forming capacity and strong barrier properties against oxygen, ultraviolet radiation, oils, and fats. These characteristics contribute to preserving food freshness and extending shelf life [19]. Although alginate films are transparent, odorless, biodegradable, and easily molded into various shapes, their poor moisture resistance and brittleness often require cross-linking or other modifications to improve mechanical strength, water vapor barrier performance, and antimicrobial functionality [1,20].

Carrageenan, which is primarily extracted from *Kappaphycus* and *Eucheuma* species of red algae, is a long-chain polysaccharide composed of consecutive disaccharide units of D-galactose and 3,6-anhydro-galactose. There are three principal types: kappa (κ), which forms firm and strong gels; iota (ι), which produces soft and elastic gels; and lambda (λ), which functions as a non-gelling thickener [21,22]. The interaction between carrageenan molecular structures and surrounding components determines its performance in diverse formulations. The presence of multiple hydroxyl and sulfate groups in carrageenan matrices facilitates the incorporation of various bioactive compounds, thereby modifying its functional properties and supporting its application as a stabilizing and texturizing polymer. Carrageenan is also widely utilized in active packaging, where essential oils or silver nanoparticles are incorporated for antimicrobial and antioxidant properties, thereby extending shelf life. Furthermore, these matrices are used to develop edible films for a range of food products [20,21].

Agars are hydrophilic colloids extracted from genera like *Gelidium* and *Gracilaria*. Though both carrageenan and agar feature 3-linked β-D-galactopyranose and 4-linked α-galactopyranose monomers, agar specifically incorporates the L-configuration for its α-galactose residues. They consist of two polysaccharides: neutral agarose and acidic agaropectin, and they are traditionally valued for their strong gel-forming properties [17,23,24]. Agar is known for its high transparency and thermoreversible gelling at low concentrations, melting at high temperatures (above 85 °C), and solidifying into stable gels when cooled (below 40 °C) [24]. It is also a physiologically inert polysaccharide with low hygroscopicity, allowing it to interact with bioactive chemicals and plasticizers to form elastic, soft gels [25]. The films exhibit moderate antibacterial activity attributed to their sulfated functional groups. However, their application is constrained by brittleness, high moisture permeability, and poor thermal stability. Incorporation of polyphenols or nanoparticles can address these limitations by enhancing hydrogen bonding and cross-linking, which in turn improves antioxidant, antimicrobial, and mechanical properties [15,23,26].

Fucoidans are complex, branched sulfated polysaccharides found in brown seaweeds’ cell and assist in moisture retention. They act as major cross-linking glycans, interlocking with cellulose scaffolds to maintain structural integrity. Structurally, they feature an *α*-L-fucopyranose backbone characterized by sulfate ester groups typically attached at the C-2, C-4, and, less frequently, at C-3 positions. Additionally, their composition frequently incorporates accessory monosaccharides such as mannose, galactose, glucose, and xylose, alongside uronic acids and acetyl groups [17,27]. These structural compositions contribute to its reported antioxidant, antimicrobial, and anti-inflammatory activities. They are used in coatings or additives in films to prevent nutrient degradation in fruits and other highly perishable food products [28]. On the other hand, ulvan is a sulfated, water-soluble polysaccharide from green seaweed made up of rhamnose, xylose, glucuronic acid, and iduronic acid [17,29]. Although less commercially developed than agar or carrageenan, it is gaining attention for its unique structures and valuable health benefits; it stands out for its metal-ion binding and low viscosity, and it is often used in composite films to boost barrier, optical, and antioxidant properties [29]. Seaweed nanocellulose is also used as a reinforcing agent to enhance the tensile strength, thermal stability, and moisture barrier of seaweed-derived films [30]. Furthermore, using residual seaweed biomass or processing by-products as sources of polysaccharides reduces raw material requirements and decreases solid waste generation [31].

### 2.2. Bioactive Compounds

Seaweeds are also an abundant source of other bioactive compounds, including polyphenols, pigments, and lipids like polyunsaturated fatty acids (PUFAs), which act as secondary metabolites with antimicrobial, antioxidant, and UV-shielding properties [5]. Polyphenols, especially bromophenols, flavonoids, and phenolic acids, constitute the majority of phenolic compounds in green and red seaweed, whereas phlorotannins are the predominant phenolic group in brown algae. Owing to their diverse biological activities, these compounds are considered relevant for active packaging formulations. When incorporated into seaweed-based films, they can delay lipid oxidation and inhibit the growth of spoilage microorganisms. Furthermore, the hydroxyl groups present in these compounds may enhance film cohesion and barrier properties by forming hydrogen bonds with polymer matrices [32,33,34].

Seaweed pigments are important secondary metabolites involved in light harvesting, photosynthesis, and photoprotection. Chlorophylls and carotenoids, such as fucoxanthin, are abundant in seaweed. They are particularly relevant in intelligent and UV-protective packaging, acting as natural UV absorbers, protecting light-sensitive food products from photo-oxidation [5,35]. Additionally, phycobiliproteins, such as phycoerythrin and phycocyanins, exhibit color-changing behavior in response to environmental factors such as pH and oxidation, which allows them to function as real-time biosensors for monitoring food freshness [36].

Although seaweeds typically contain a low total lipid content (less than 5%), they are a significant source of PUFAs such as omega-3 (EPA and DHA) and omega-6 (linoleic acid) [5]. Seaweed-derived lipid extracts and lipid-mediated nanoparticles exhibit effective moisture barrier properties, facilitate hydrophobic surface modification, and improve moisture resistance, thereby broadening their suitability as composite film coatings or additives. Additionally, their inherent antioxidant and antimicrobial properties enable active packaging performance [20,37].

### 2.3. Extraction of Seaweed Polysaccharides and Bioactive Compounds

To enhance the isolation of seaweed-derived polysaccharides and other bioactive compounds, pre-treatments are commonly applied to algal biomass before extraction. These pre-treatments help prevent the co-extraction of unwanted compounds with similar solubility and break down cell walls to improve the transfer of target compounds into the extraction solvent. Because seaweed cell walls are complex, effectively disrupting these structures is a prerequisite for releasing high-purity bioactive molecules [38,39]. Preparation of seaweed includes washing and cleaning, preferably with distilled water, to remove salt and other impurities such as sand, sediments, and debris, as well as drying to reduce moisture content for preservation and quality control. Freeze-drying is often preferred because it preserves the integrity of heat-sensitive biomolecules and can lead to higher extraction yields. Mechanical grinding or milling the seaweed into fine powders (typically 0.25–1 mm) increases the surface-to-volume ratio. Finer particles enable better contact between the biomass and the extraction solvents, significantly enhancing mass transfer and extraction efficiency [39,40].

Chemical pretreatments are also used to eliminate non-target compounds that may interfere with the primary extraction or contaminate the final product. Solvents such as ethanol, acetone, or chloroform/methanol mixtures are used to remove lipids, chlorophylls, and other hydrophobic pigments, while high polarity molecules like protein and minerals are extracted in water [41,42]. On the other hand, to prevent polyphenols from contaminating target polysaccharides (like alginate or fucoidan), seaweed is often soaked in formaldehyde. Formaldehyde causes phenols to polymerize into insoluble high-molecular-weight components, which are then easily separated from the liquid extract [40,41,43]. Acid or alkali pre-treatment is a common method used. Dilute acids, mainly HCl, have been used to remove non-target compounds present in the seaweed, such as polyphenols and easily degradable polysaccharides like fucoidans and laminarins, and to eliminate polyvalent cations such as Ca^2+^ and Mg^2+^ [43,44]. Alkali pre-treatment can be used to degrade cell walls specifically to enhance the gelling properties of hydrocolloids like agar [43]. Various processing methods, such as chemical and enzymatic hydrolysis and fermentation, biochemically modify the seaweed substrate to enhance flavor, safety, nutritional bioavailability, and functional properties like antioxidant capacity [45,46,47,48].

**Figure 3:**
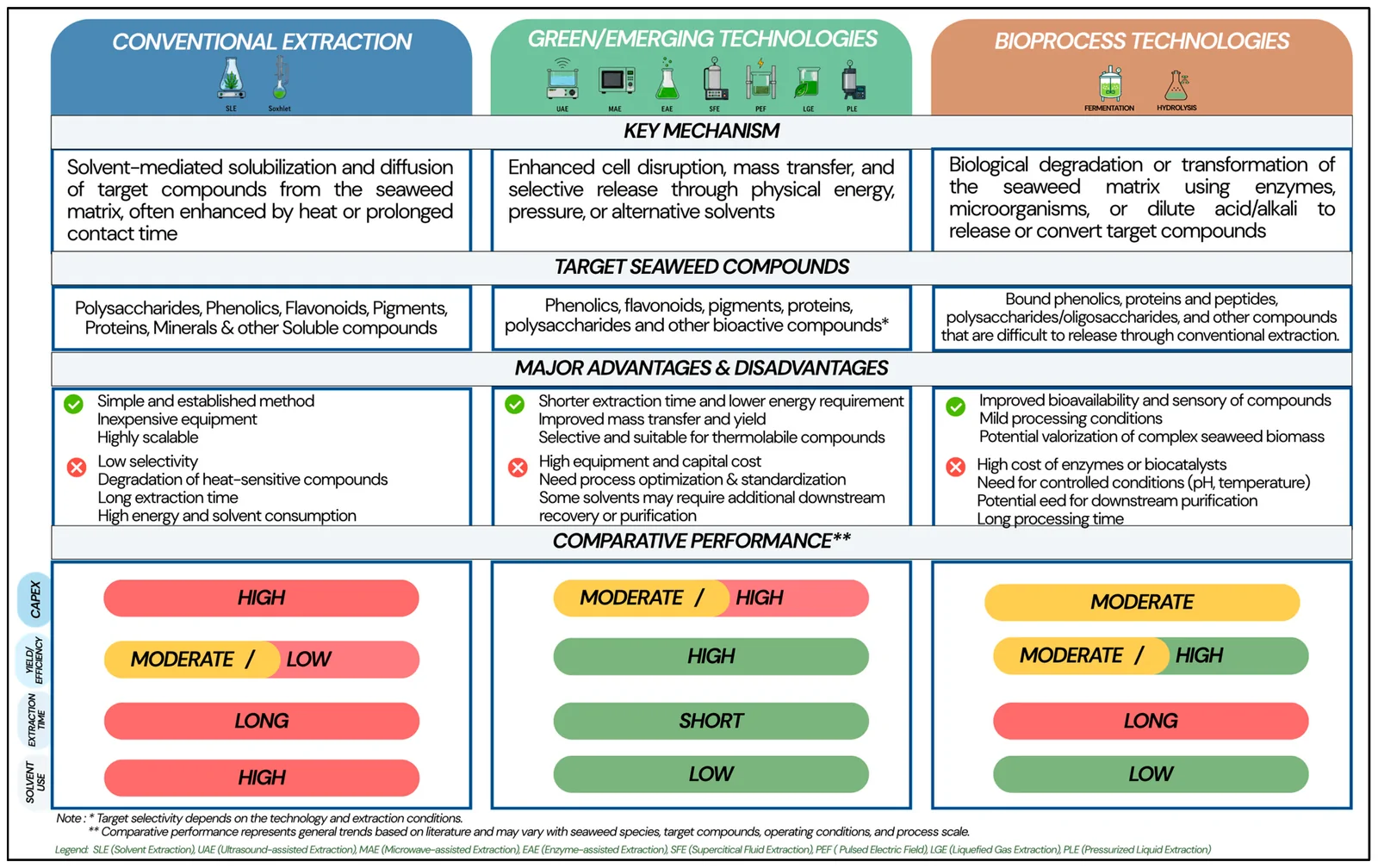
Conventional, emerging/green, and bioprocessing strategies for the extraction of seaweed-derived bioactive compounds [38,39,45,46,47,48,50,51,52,53].

Extraction methods generally fall into two main categories: traditional techniques, such as solvent (solid–liquid) extraction and Soxhlet extraction, and advanced or green technologies, which include accelerated solvent extraction, ultrasound-assisted extraction, microwave-assisted extraction, pressurized liquid extraction, supercritical fluid extraction, enzyme-assisted extraction, and extraction with biomolecule solvents like NADES [39,49]. Conventional extraction methods remain central to industrial seaweed processing due to their scalability. However, emerging green technologies offer notable improvements in sustainability and efficiency. Each technique operates under different parameters and is suited to specific compounds or bioactives. The optimal method depends on the target compound and available equipment or solvents [39]. Figure 3 compares the advantages, limitations, and target compounds of both conventional and emerging extraction technologies. Conventional extraction techniques typically involve treating seaweed biomass with various solvents, such as hot water or acidic or salt solutions, at high temperatures for several hours (1–48 h). While simple and requiring low investment, it is labor-intensive and energy-consuming, often resulting in low selectivity and high levels of impurities [43,50]. In contrast, green extraction technologies, including ultrasound-, microwave-, enzyme-, and pulsed-electric-field-assisted extraction, can enhance mass transfer and extraction efficiency while reducing solvent use, processing time, and energy consumption, with greater potential to preserve the functional properties of heat-sensitive compounds [38,39,50,51,52].

Extraction techniques further contribute to variability in chemical composition, bioactivity, and efficiency of seaweed-derived compounds. These variations can result in differences in extract yield, potency, and quality, complicating reproducibility across batches and comprehensive analysis [54,55]. Choosing the right extraction method to obtain beneficial compounds from seaweed is important and depends on the specific compounds being targeted. Factors to consider include the extract’s polarity, the compound’s thermal stability, the expected yield, available equipment, and the intended use of the extract [39,49]. Modern biorefinery approaches often combine two techniques to maximize efficiency. For example, Ultrasound-Microwave-Assisted Extraction (UMAE) uses microwaves for rapid heating and ultrasound to enhance mass transfer through cavitation, resulting in higher extraction efficiency, reduced processing time, energy savings, and fewer by-products compared to using either technique alone [38].

## 3. Techno-Functional Properties of Seaweed-Based Polymers for Seafood Packaging

The effectiveness of seaweed-based packaging is determined by its physical, mechanical, barrier, and functional properties, which determines its ability to protect seafood from major factors responsible for quality deterioration, thus maintaining food quality and safety and extending shelf life. Food packaging materials must provide adequate barriers to oxygen, moisture, and other gases, preventing spoilage, oxidation, and microbial growth. Additionally, these materials require sufficient mechanical stability to endure transportation and storage and to inhibit the migration of harmful substances into food products. Other important properties, including optical characteristics, thermal stability, and biological functionality, further influence packaging performance depending on the intended application [2,6].

### 3.1. Mechanical Properties

The mechanical integrity of seafood packaging films is critical for ensuring their ability to withstand processing, transportation, and storage without tearing, puncturing, or permanent deformation. Tensile strength (TS), Young’s modulus (YM), and elongation at break (EAB) serve as key indicators of packaging performance. Tensile strength (TS), elongation at break (EAB), and Young’s modulus (YM) are primary indicators of the mechanical performance of packaging materials, specifically film strength, flexibility, and stiffness, respectively [56]. Tensile strength refers to the maximum tension a film can withstand before breaking, which reflects the mechanical strength (rigidity) of the film, whereas elongation at break denotes the greatest elongation of the film prior to rupture, expressed as a percentage (%EAB), which reflects the ductility (flexibility) of the film [57]. Materials with high tensile strength but limited flexibility may crack during handling, whereas highly extensible films lacking adequate strength may fail to protect packaged food effectively [2,22]. Commercial food packaging typically requires a tensile strength exceeding 7 MPa and an elongation at break above 40%, although optimal values vary depending on the specific food application [6]. Conventional petroleum-based plastics such as polyethylene terephthalate (PET) and the bioplastic polylactic acid (PLA) demonstrate tensile strengths of 55–75 MPa and 50–70 MPa, respectively [58,59].

Seaweed-based films exhibit considerable variation in mechanical performance depending on polymer structure and formulation. Alginate-based films demonstrate considerable mechanical strength and flexibility, attributes that can be further enhanced through ionic cross-linking to increase tensile strength and durability [19,60] Ureña et al. [61] evaluated alginates with different molecular weights and mannuronic-to-guluronic acid ratios and reported that the resulting films exhibited a tensile strength of approximately 42.5 MPa, Young’s modulus of approximately 1.9 GPa, and elongation at break of approximately 8.4%. On the other hand, pure ι-carrageenan and κ-carrageenan films have been reported to exhibit tensile strengths of 18.36 and 42.5 MPa, respectively, with elongation at break values of 9.86% and 3.9%, respectively [62,63]. Carrageenan films are naturally brittle and generally require the addition of plasticizers, other biopolymers, or nanoparticles to improve mechanical strength and flexibility [21,64]. Agar produces relatively strong and flexible films; however, its inherent fragility and low elongation at break often necessitate blending with other biopolymers or cross-linking agents to improve structural durability [65,66].

### 3.2. Barrier Properties

Barrier properties, such as resistance to gases, moisture, and UV light, are essential in food packaging to extend shelf life and preserve product quality. Water vapor permeability (WVP), oxygen permeability or oxygen transmission rate (OTR), moisture content, water solubility, swelling capacity, and water absorption are evaluated to determine the film’s ability to resist moisture transfer and gas diffusion, which are critical factors for maintaining the shelf life of high-moisture foods like seafood. Insufficient barrier protection can lead to rapid spoilage, microbial growth, and loss of nutritional and sensory attributes [2,19,22].

Water vapor permeability (WVP) is the most extensively studied barrier property. WVP measures the amount of water that permeates per unit area and time (g/m·s·Pa). The primary goal in film development is to achieve low WVP values to minimize moisture transfer between food products and the surrounding atmosphere [57]. Seaweed–polysaccharide films typically demonstrate limited water-vapor barrier performance compared to conventional petroleum-based polymers, largely due to the hydrophilic nature of polysaccharides such as alginate, carrageenan, and agar. Reported WVP values vary significantly depending on polymer type, molecular structure, film thickness, relative humidity, plasticizer content, crosslinking, and processing conditions. For instance, pure ι-carrageenan films have been reported to exhibit a WVP of 40.58 × 10^−11^ g·m·Pa^−1^·m^−2^·s^−1^ [62], whereas agar films generally exhibit higher water-vapor permeability than commercial polymers such as PET and PP and other biopolymers such as PLA [67]. Similarly, alginate exhibits a prominent hydrophilic nature, which contributes to its relatively poor resistance to water-vapor permeation; water-vapor permeability values exceeding 7.8 × 10^−8^ mol·m^−2^·s^−1^·Pa^−1^ have been reported even at low relative-humidity conditions [61].

Seaweed-based films provide effective selective physical barriers against oxygen and carbon dioxide, which are critical for suppressing oxidative spoilage and maintaining seafood quality [68,69]. While their inherent hydrophilicity often results in poor moisture resistance, these barrier properties can be significantly improved through chemical cross-linking or by utilizing advanced bilayer and layer-by-layer (LbL) architectures that establish dense molecular networks [21,70]. The incorporation of hydrophobic additives like essential oils or lipid-derivative extracts further mitigates water vapor transfer by increasing the matrix’s surface hydrophobicity [71,72]. Additionally, integrating reinforcing nanofillers, such as titanium dioxide or metal-organic frameworks, further enhances barrier properties by restricting gas and vapor diffusion [73,74].

### 3.3. Optical and Thermal Properties

The optical and thermal properties of seaweed-based films affect both processing efficiency and consumer acceptance. Most seaweed films, including agar and alginate, exhibit high transparency, which facilitates visual inspection of packaged products. In contrast, adding pigments, phenolic extracts, or nanoparticles typically increases opacity but enhances functional properties [19,26]. On the other hand, thermal properties are crucial for food packaging materials, as they affect how well the packaging performs under temperature changes during processing or storage. Seaweed-derived films provide adequate thermal stability for most food applications but are less heat resistant than petroleum-based plastics. For example, carrageenan films remain stable up to 200–210 °C [21,22,69].

### 3.4. Antioxidant, Antimicrobial, and UV Protective Properties

Seaweed-derived polysaccharides have emerged as promising materials for active packaging due to their inherent antimicrobial and antioxidant properties, primarily associated with the presence of sulfated polysaccharides, polyphenols, pigments, and other bioactive metabolites found in seaweeds. Integrating these compounds into packaging films enables active packaging systems that can delay oxidation, inhibit microbial proliferation, and extend the shelf life of food products. Notably, fucoidan exhibits significant antibacterial activity against prevalent foodborne pathogens, as well as strong free radical scavenging, metal-chelating, and lipid peroxidation inhibitory activities [70,75]. For example, fucoidan has not only improved the barrier and mechanical properties of chitosan films but also enhanced antimicrobial and antioxidant properties, thus prolonging the shelf life of fish (salmon) during cold storage [29].

Ultraviolet (UV) radiation, particularly UV-A (315–400 nm) and UV-B (280–315 nm), can promote photooxidation and degradation of sensitive food components, resulting in changes in color, nutrients, flavor, and overall freshness. Therefore, UV-barrier packaging is important for reducing light-induced deterioration and maintaining seafood quality during storage, with studies demonstrating that biopolymer-based films can provide UV shielding through absorption, reflection, or scattering of UV radiation [76,77]. Additionally, seaweed films offer natural UV-light shielding due to inherent photoprotective compounds like phenols and flavonoids, which protect light-sensitive nutrients from photodamage [78,79]. Furthermore, although alginate, agar, and κ-carrageenan possess limited intrinsic biological activity, they also serve as excellent carriers for other natural antioxidants and antimicrobial agents such as plant polyphenols, essential oils, nanoparticles, and anthocyanins, further enhancing the performance of the film [74].

**Figure 4:**
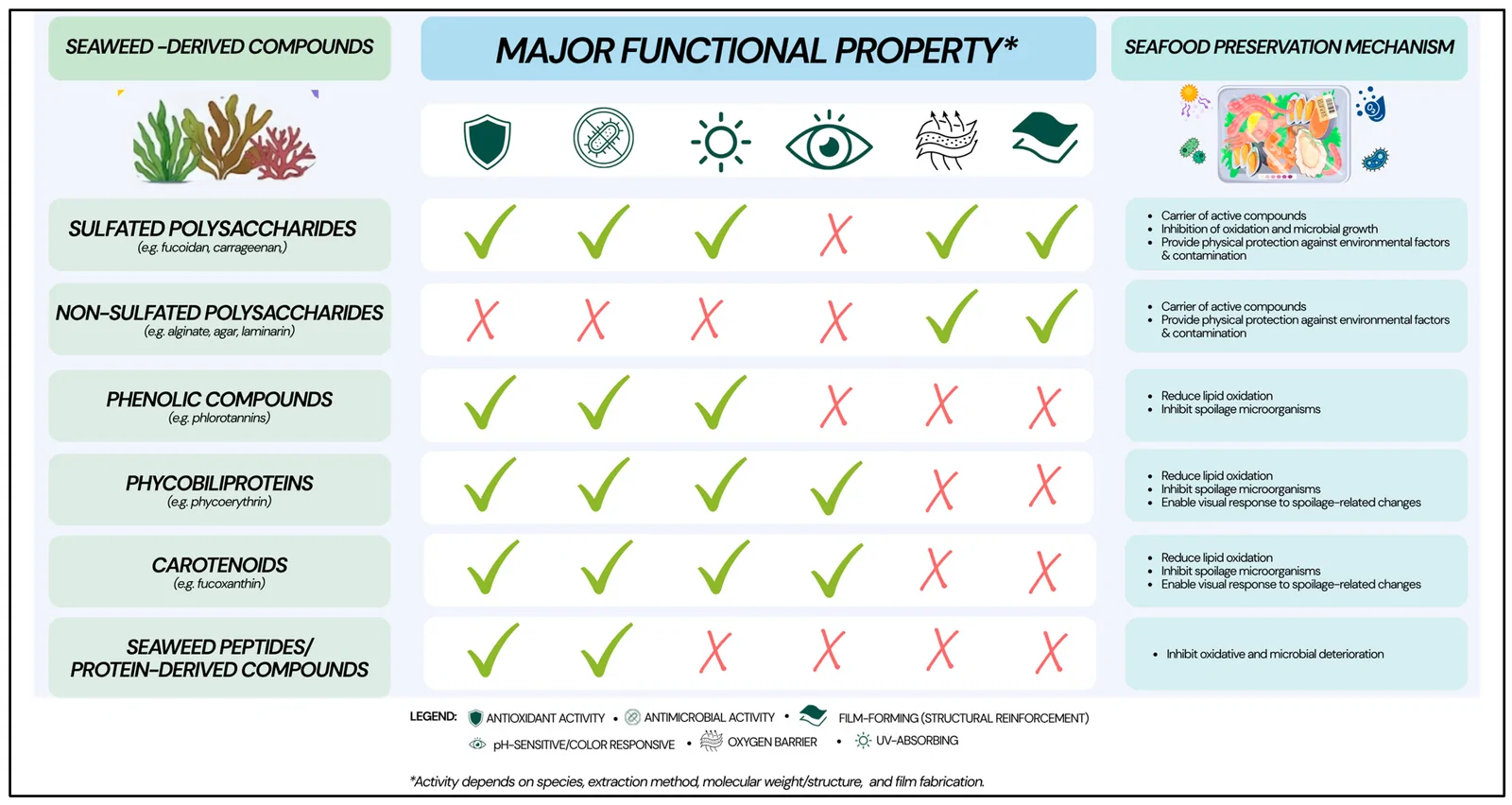
Functional properties of seaweed-derived bioactive compounds and their mechanisms in seafood preservation and freshness monitoring [11,15,19,36,62,80,81].

In summary, seaweed-derived polysaccharides provide structural and barrier functions, while other bioactive compounds impart antioxidant, antimicrobial, and, in some cases, pH-responsive properties to packaging systems (Figure 4). These properties may help delay lipid oxidation and inhibit microbial spoilage when incorporated into seafood packaging matrices. As a result, seaweed-based packaging can function as both a passive barrier and an active packaging system [11,15,19,36,62,80,81]. These inherent functional properties provide the basis for their use in active and smart packaging, with their applications in seafood preservation and freshness monitoring discussed in the following section.

## 4. Applications of Seaweed-Based Packaging in Seafood Preservation

### 4.1. Seafood Spoilage Mechanisms and Freshness Indicators

Seafood, including fish, crustaceans, mollusks, and aquatic products, are nutrient-dense food characterized by high-quality proteins, essential amino acids, polyunsaturated fatty acids, particularly omega-3 fatty acids, vitamins, minerals, and a high moisture content, with its biochemical composition varying considerably among species [9]. These products are highly perishable and undergo rapid biochemical, chemical, and microbiological deterioration following harvest, resulting in lipid and protein oxidation, autolysis, microbial proliferation, and the accumulation of total volatile basic nitrogen (TVB-N), trimethylamine (TMA), ammonia, and other biogenic amines resulting in undesirable off-flavors and odors [9,10].

Microbial spoilage is a major cause of seafood quality deterioration, with psychotropic bacteria such as *Pseudomonas* and *Shewanella* spp., degrading proteins, lipids, and nitrogenous compounds, resulting in the production of TMA, ammonia, sulfides, biogenic amines, and other volatile metabolites that cause off-odors, discoloration, and slime formation. The accumulation of these spoilage metabolites, particularly volatile basic compounds, quantified as TVB-N, is a key indicator of seafood freshness and spoilage [8,10]. TVB-N is widely recognized as a chemical indicator of seafood freshness because it reflects the accumulation of volatile nitrogenous compounds generated during microbial and enzymatic degradation [82]. In addition, total viable count (TVC) or aerobic plate count (APC) is routinely used to assess microbial quality and spoilage in seafood, with 7 log CFU/g commonly considered the upper microbiological acceptability limit [83]. Additionally, bacteria associated with seafood contamination, including *Escherichia coli*, *Staphylococcus aureus*, *Campylobacter jejuni*, *V*. *parahaemolyticus*, and *L. monocytogenes*, are particularly relevant [8,84].

Enzymatic and chemical deterioration are important mechanisms of seafood spoilage that occur in parallel with microbial degradation. Enzymatic deterioration, also known as autolysis, results primarily from endogenous enzymes in seafood tissues, particularly proteases and lipases, which remain active post-harvest. Proteases catalyze the hydrolysis of muscle proteins into peptides and amino acids, leading to softening and loss of textural integrity. Lipases hydrolyze lipids into free fatty acids, which may subsequently undergo oxidation [1,10]. In crustaceans such as shrimp, endogenous polyphenol oxidase (PPO) promotes melanosis, which is characterized by the formation of dark pigments or black spots on the shell. Although melanosis is not necessarily associated with microbial spoilage or safety concerns, it substantially diminishes product appearance and consumer acceptability [9,85]. Chemical deterioration, meanwhile, primarily results from lipid oxidation, especially in seafood high in polyunsaturated fatty acids. Exposure to oxygen, light, and other pro-oxidative conditions promotes the formation of lipid hydroperoxides, which subsequently decompose into aldehydes, ketones, and other compounds that cause rancid odors and flavors. Primary lipid oxidation is typically assessed using peroxide value (PV), while thiobarbituric acid reactive substances (TBARS), particularly malondialdehyde, serve as indicators of secondary lipid oxidation [86]. Protein degradation and the accumulation of volatile nitrogenous compounds are evaluated through TVB-N and TMA. Additionally, changes in color, texture, odor, and sensory acceptability provide complementary indicators of seafood quality deterioration [87,88].

Packaging therefore plays a critical role in protecting seafood from adverse environmental factors such as heat, oxygen, light, and contamination, thereby maintaining product quality and extend shelf life. Understanding these spoilage mechanisms and freshness indicators provide the basis for evaluating how seaweed-based packaging can delay seafood deterioration through protective and active functions, enabling real-time freshness monitoring and thus improve the overall performance of the films [9,10].

### 4.2. Seaweed-Based Packaging Systems for Seafood Preservation and Freshness Monitoring

For the purpose of this review, seaweed-based packaging systems are broadly classified according to the form and primary function of the seaweed-derived material incorporated into the packaging; (i) seaweed polysaccharide-based films and coatings, in which alginate, carrageenan, agar, or other seaweed polysaccharides serve as the principal seaweed-derived matrix component, either alone or in combination with natural biopolymers (e.g., chitosan, gelatin, starch, or cellulose), synthetic or modified polymers such as polycaprolactone (PCL), polyhydroxyalkanoates (PHA), polylactic acid (PLA), carboxymethyl cellulose (CMC), or other polymeric additives, which modify the functional properties of the packaging; (ii) packaging systems incorporating isolated seaweed-derived bioactive compounds, such as phenolics, carotenoids, phycobiliproteins, and fucoidan, which provide active antioxidant, antimicrobial, or other functional properties; and (iii) whole seaweed extract-based systems, in which complex mixtures of seaweed constituents are incorporated to provide multifunctional activity.

Seaweed polysaccharide matrices provide film-forming and barrier properties that can reduce moisture and oxygen transfer, whereas compounds such as fucoidan may provide antioxidant and antimicrobial activities [89,90]. Crude seaweed extracts, which contain complex mixtures of polysaccharides, phenolic compounds, pigments, and other bioactive constituents, may provide combined antioxidant and antimicrobial effects, which could improve the film’s functional properties [72,91,92]. These functions are particularly relevant to seafood, where microbial growth, lipid oxidation, and protein degradation occur simultaneously. Thus, preservation may result from intrinsic barrier properties, the antioxidant or antimicrobial activities of isolated compounds or whole extracts, or their combined effects [35,81,91]. In contrast, pH-responsive compounds such as phycoerythrin and other pigments primarily serve a smart packaging function by enabling visual freshness monitoring rather than directly preserving the product [80]. Multifunctional systems may integrate barrier, active, and smart functions within a single packaging architecture. The packaging applications and preservation or functional outcomes of these systems are discussed in the following subsections on edible films and coatings, active packaging, smart packaging, and emerging multilayer and multifunctional systems. Comparative data presented in Table 1 further demonstrate that the effectiveness of these seaweed-based packaging systems in seafood preservation varies significantly, depending on the specific polymer matrix and incorporated functional additives.

#### 4.2.1. Edible Films and Coatings

Edible films and coatings remain the most established application of seaweed-derived polymers for seafood preservation, both of which function as effective moisture and gas barriers. Edible coatings are deposited directly onto the food matrix to create a protective layer that mitigates dehydration, oxidative degradation, and microbial spoilage, whereas edible films are pre-fabricated, standalone matrices designed to encase the food product, thereby controlling mass transfer and preserving product quality attributes [15,104].

Numerous studies have demonstrated that seaweed-based edible coatings effectively preserve seafood quality by enhancing gas barrier properties, reducing moisture loss, and maintaining texture during chilled storage. Specifically, alginate-based edible films or coatings have attracted considerable attention for their ability to extend the shelf life of seafood products. Incorporating proteins or other polysaccharides into these films further improves their structural integrity and mechanical strength. The inclusion of nanoparticles or natural antioxidants, such as silver nanoparticles, zinc oxide, phycocyanin, or fucoidan, imparts additional antimicrobial and antioxidant effects. These modifications slow spoilage by inhibiting microbial growth and oxidation, thereby reducing quality loss indicators such as TVB-N, TMA, TBA, and melanosis. Overall, such coatings or films significantly extend seafood shelf life and quality [35,78,94].

#### 4.2.2. Active Packaging

Active packaging refers to materials that physically or chemically interact with food or the packaging environment to maintain the safety and quality of food products. Such systems are engineered to reduce respiration rates, inhibit microbial growth and lipid oxidation, limit moisture migration, and provide ultraviolet protection [19,74,105]. Seaweed-based polymers like alginate, agar, and carrageenan are often enriched with antioxidants (e.g., fucoidan, phlorotannins, or essential oils) to scavenge free radicals and inhibit lipid oxidation, and antimicrobial agents (e.g., Ag, ZnO, TiO_2_ nanoparticles or plant extracts) to disrupt the cell membranes of dominant spoilage bacteria, thus extending the shelf life of fish and other seafood products [105,106]. The composite films developed showed enhanced tensile strength together with excellent antioxidant, UV-blocking, or antibacterial properties. Antioxidants such as phycocyanin or fucoidan delay the accumulation of TVB-N and TMA, slowing the protein degradation and lipid oxidation that cause rancidity and off-flavors in fish. They are also often used as carriers for nano-fillers (ZnO, AgNPs, carbon dots (CDs)), or metal–organic frameworks (MOFs) that deactivate bacterial DNA reproduction, effectively extending the microbial shelf life of seafood products by several days [68,79,91,107].

#### 4.2.3. Smart Packaging

Smart packaging incorporates indicators or sensors to enable real-time monitoring of food quality, freshness, and safety by detecting changes in pH, temperature, moisture content, or chemical composition. These systems produce actionable signals, often observed as color changes, which facilitate the assessment of product integrity by manufacturers, logistics providers, and consumers [100,108]. Most seaweed-based intelligent films utilize halochromic sensors and natural indicators or pigments like anthocyanins, phycoerythrins, curcumin, and alizarin, which undergo distinct color shifts (e.g., yellow to red or pink to blue) as volatile nitrogenous compounds (ammonia, TMA) accumulate during protein degradation, allowing for non-destructive monitoring of highly perishable products like seafood [96,97,103]. During seafood storage, microbial metabolism and enzymatic degradation generate ammonia, TMA, TVB-N, and biogenic amines. These accumulated alkaline gases dissolve in the film moisture, raising the internal pH and triggering a visible color shift in embedded natural pigments. These biochemical changes provide reliable indicators for freshness monitoring [74,97,100,108].

Feng et al. [73] developed an intelligent active film by embedding ammonia-sensitive, antibacterial cobalt-based MOF (Co-MOF) nanoparticles into a seaweed-derived sodium alginate (SA) matrix. This seaweed-based film exhibited enhanced mechanical strength, thermal stability, UV shielding, and water vapor barrier properties alongside strong antibacterial activity. Notably, the composite film demonstrated effective real-time spoilage monitoring by changing color from pale pink to brownish black when exposed to ammonia during shrimp degradation. Similarly, roselle anthocyanin incorporated into an agar/Polyvinyl alcohol (PVA) indicator film effectively monitored salmon freshness at 4 °C by shifting visually from red to green in response to detecting spoilage amines like ammonia, TMA, and DMA [101]. Moreover, Ali et al. [80] incorporated the seaweed-derived pigment phycoerythrin (PE) into a sodium alginate (SA)-based film reinforced with cellulose nanofibrils (CNF) and fish gelatin to develop a smart packaging system for salmon freshness monitoring. The SA, gelatin, and CNF matrix provided the structural and protective properties of the film, while PE served as the pH-responsive indicator. As seafood spoilage progressed, the increase in pH associated with microbial and biochemical deterioration induced a distinct color transition of the film from red to purple–brown and eventually colorless. These color changes corresponded with increases in bacterial counts and TVB-N, demonstrating the potential of PE to provide a visual, real-time indication of salmon freshness. Thus, in this system, the primary preservation function is associated with the film matrix, whereas PE primarily contributes the smart monitoring function rather than directly delaying spoilage.

#### 4.2.4. Multi-Functional and Multi-Layer Packaging Systems

Multilayer packaging systems are being investigated to overcome the moisture sensitivity and poor mechanical strength of single-layer seaweed-derived films. Rather than relying on a single polymer, these systems combine multiple functional layers, each designed to perform a specific role such as moisture resistance, oxygen barrier protection, mechanical reinforcement, active preservation, or freshness monitoring [70,102,103]. Typically, a hydrophilic seaweed-derived layer composed of alginate, agar, or carrageenan provides effective oxygen-barrier properties and serves as a carrier for antioxidants or antimicrobial compounds. In contrast, an outer hydrophobic or relatively less hydrophilic layer composed of complementary biopolymers, such as chitosan, PCL, PHA, PLA, or bacterial cellulose (BC), can reduce water-vapor transmission and improve moisture resistance [102,103,109]. The incorporation of proteins, lipids, or other complementary polymers can further modify the mechanical and barrier properties of seaweed-based films [69,109]. For example, alginate–whey protein films containing curcumin exhibited enhanced hydrophobicity, antioxidant activity, UV-blocking capacity, and reduced water-vapor transmission demonstrating the potential of combining different components to improve film functionality [109]. Similarly, layer-by-layer (LbL) systems involve the sequential application of oppositely charged polyelectrolytes, such as polycationic chitosan and polyanionic alginate, which significantly improve the film barrier properties for water vapor and gases. These systems create a dense network that controls the release of active agents like fucoidan that exhibit antimicrobial or antioxidant activity. Their release can provide an additional preservation effect by suppressing microbial growth and oxidative deterioration effectively extending the shelf life of seafood products [70,109]. In fish fillet preservation, Khorami et al. [70] demonstrated that a chitosan–alginate LbL coating containing fucoidan extended the shelf life of rainbow trout fillets from 6 to 16 days and reduced total viable counts, psychrotrophic bacteria, lactic acid bacteria, TVB-N, and TBARS compared with control treatments. These results indicate that preservation can result from the combined contribution of the multilayer barrier structure and the synergistic effects of the alginate/fucoidan/chitosan.

More recently, researchers have incorporated active antimicrobial layers together with intelligent colorimetric indicator layers within the same packaging structure, enabling simultaneous preservation and freshness monitoring. These systems can help delay microbial proliferation, protein degradation, lipid oxidation, and associated quality deterioration in seafood, while the incorporation of indicator compounds can provide an additional function for real-time freshness monitoring [70,74,102,103]. These multifunctional systems more closely resemble commercial multilayer plastic packaging while retaining the environmental advantages of biodegradable materials [74,103]. For example, Khan et al. [79], developed carrageenan-based active and smart packaging films containing Zn–CDs and anthocyanin. These composite films demonstrated excellent UV shielding, antioxidant performance, and antimicrobial properties that contributed to the suppression of microbial proliferation, mitigating spoilage, and extending product shelf life, while the anthocyanin-based color response enabled real-time monitoring of shrimp freshness.

## 5. Food Safety and Regulatory Considerations

Food safety and regulatory compliance are critical considerations in the development and commercialization of seaweed-derived packaging materials for food applications [74,110]. Although morbidity and mortality associated with seaweed consumption are rare, concerns regarding their potential toxicity persist among researchers and regulatory authorities [111]. The biological properties of seaweed and its growth environment can introduce specific risks that require comprehensive monitoring and standardized management [111,112].

The main seaweed polysaccharides used in food packaging, agar, alginate, and carrageenan have been extensively utilized as food additives and are generally recognized as safe (GRAS) by the US Food and Drug Administration (USFDA). Agar has undergone comprehensive toxicological, mutagenic, and teratogenic evaluations with no reported adverse effects during decades of commercial use, while alginate and carrageenan are similarly regarded as safe for food-contact applications because of their biodegradability, biocompatibility, and established safety profiles [113,114]. However, seaweeds have the potential to naturally bioaccumulate contaminants such as heavy metals, including arsenic, cadmium, lead, and mercury, pesticides, microplastics and other persistent organic pollutants (POPs), as well as the relatively high iodine content of some seaweed species [11,12]. Excessive and prolonged intake of these compounds can pose serious health risk such as carcinogenicity, neurotoxicity, cardiovascular diseases, and oxidative damage, thus the use of seaweed in packaging systems necessitates careful raw material selection, post-harvest treatments, purification, and routine contaminant monitoring [112].

Seaweed products present potential allergenicity risks, particularly for whole seaweed biomass or crude extracts, due to cross-contact with marine-derived allergens such as crustacean allergens (e.g., tropomyosin) during cultivation, harvesting, and processing. These allergens can be introduced by organisms such as amphipods or through co-cultivation with other marine species, thereby posing risks to individuals with crustacean or shellfish allergies [115,116]. Microbiological contamination is also a significant safety concern for seaweed-derived food-contact materials, as seaweeds can harbor microorganisms from their cultivation environment and may become contaminated or recontaminated during harvesting, handling, and processing. Pathogenic microorganisms, including *Bacillus*, *Vibrio*, *Aeromonas*, *Escherichia coli*, *Salmonella*, and *Listeria monocytogenes*, have been reported in association with seaweed products, highlighting the need for stringent hygienic handling and processing controls [117,118]. Furthermore, seaweeds may contribute to the development and dissemination of antimicrobial resistance (AMR), as their surfaces support dense microbial populations and may facilitate horizontal transfer of antimicrobial resistance genes. Resistant bacteria may also be introduced during harvesting, transport, or processing. Although direct evidence linking edible seaweed products to AMR transmission remains limited [117], appropriate processing and preservation strategies, such as washing, drying to reduce water activity, thermal treatment, fermentation, salting, irradiation, and high-pressure processing, can reduce microbial loads and enhance the microbiological safety of seaweed materials [117,118].

European Union (EU) regulations require that food contact materials do not transfer constituents at levels that could endanger human health or alter food quality [6]. Incorporating functional additives, including nanoparticles, essential oils, antioxidants, antimicrobial agents, and colorimetric indicators, introduces additional regulatory requirements related to migration behavior and toxicological safety. A detailed analysis of the migration behavior of different active components from seaweed-based packaging materials and how those components influence the specific characteristics of the food items should be evaluated in accordance with applicable food contact requirements [26].

Ensuring consistent safety and performance require standardized analytical and quality-control procedures throughout the production chain. Harmonized sample preparation, reference materials, and inter-laboratory validation reduce variability in the reported biochemical composition of seaweed materials [119,120]. Routine testing for contaminants, microbial hazards, and other undesirable constituents prior to incorporation into packaging is essential for achieving batch-to-batch consistency. Furthermore, comprehensive regulatory standards and legislation are necessary for the development and commercialization of seaweed-based packaging. Future initiatives should therefore prioritize harmonized quality standards, contaminant-monitoring protocols, labeling requirements, migration testing, and safety-assessment frameworks for emerging seaweed-derived food-contact materials [111,121]. Currently, there is no harmonized international framework or standard that specifically addresses global seaweed food safety to ensure consistent safety, quality, and responsible commercialization of seaweed-derived products [122,123]. In response, the Codex Alimentarius Commission is actively developing global safety and quality standards for seaweed to promote regulatory consistency, protect consumers, and support fair trade [123].

## 6. Challenges and Future Perspectives

Despite the significant progress in developing seaweed-derived packaging materials, the transition from promising laboratory research to commercially viable products remains constrained by technical and economic limitations. Table 2 compares major packaging materials across key parameters, including performance on seafood preservation, cost, biodegradability, processing, and scalability, to highlight the techno-economic gaps seaweed-based systems must bridge to compete with conventional and other bio-based materials.

The comparative analysis illustrates that no single packaging material achieves optimal mechanical performance, barrier properties, low cost, biodegradability, and scalability simultaneously. Conventional petroleum-based plastics, including polyethylene (PE), polypropylene (PP), and polyethylene terephthalate (PET), exhibit superior mechanical and barrier properties. These materials have been refined over decades for high-throughput manufacturing, resulting in consistent performance at relatively low cost. However, their reliance on fossil resources, limited biodegradability under natural conditions, and environmental persistence are significant drawbacks [125,128].

Seaweed-based materials offer advantages such as resource abundance, rapid growth, and biodegradability. However, seaweed-derived films are typically highly hydrophilic, leading to poor water vapor resistance, reduced mechanical strength in humid environments, and limited long-term stability compared with conventional plastic packaging and hydrophobic polymers such as PLA, which generally provide more effective moisture barriers. This limitation is particularly relevant for packaging high-moisture foods, including seafood. To mitigate the performance limitations of seaweed-derived films, polymer blending, nanocomposite reinforcement that creates physical barriers to permeability, chemical or ionic crosslinking, and multilayer film designs have substantially improved these properties. However, further optimization is needed to achieve the mechanical durability and barrier performance required for industrial food packaging [6,65,100]. At the same time, future research must evaluate long-term storage stability, perform migration studies, safety and life cycle assessments, and satisfy the regulatory requirements for food-contact materials [26].

Starch- and cellulose-based films present a similar trade-off to seaweed-based materials. These materials are inexpensive, widely available, and can be readily processed into films or thermoplastic products, which makes them attractive for cost-effective and scalable applications. However, like seaweed, they are highly hydrophilic and may exhibit brittleness and poor moisture resistance [125,126]. In comparison, seaweed offers both structural polymers and naturally occurring bioactive compounds, enabling the development of packaging systems that integrate structural and active functions. These functional properties are particularly desirable for seafood applications [6].

Economic feasibility is also a primary challenge in seaweed-based packaging. The raw seaweed biomass itself may be relatively inexpensive or locally abundant, particularly when cultivated species, processing residues, or underutilized biomass streams are considered. The cost associated to extracting and purifying high-quality polysaccharides and bioactive compounds often require multiple processing steps, specialized equipment, large volumes of chemicals or water, and substantial energy consumption, all of which increase production costs. These costs are further increased by the incorporation of plasticizers, crosslinking agents, nanofillers, and bioactive compounds needed to improve film performance [3,129]. The production costs for seaweed-based bioplastics are currently estimated to be much higher than those of petroleum-derived counterparts, sometimes as much as twice the price [23].

In addition, the chemical composition and functional properties of seaweed hydrocolloids vary considerably with species, geographic origin, cultivation conditions, harvesting season, and extraction methods, resulting in inconsistencies in film quality and processing performance. Such variability constitutes a significant challenge for industrial production, where uniform raw materials and reproducible product properties are essential [6,11]. Furthermore, most studies have been conducted at the laboratory scale and in short-term storage trials, with limited evaluation under pilot-scale manufacturing or commercial processing and cold-chain conditions. Scaling up these technologies will require the development of cost-effective, green extraction processes, continuous manufacturing techniques, and standardized fabrication protocols capable of producing packaging materials with consistent quality and performance [68,69,129]. This review demonstrates that the main challenge for seaweed-based packaging, particularly in seafood preservation, involves more than simply replicating the performance of conventional plastics. Future research should focus on optimizing the balance among film performance, active functionality, cost, scalability, and environmental impact.

## 7. Conclusions

Seaweed-derived packaging materials are promising sustainable alternatives to conventional plastics because of their renewable nature, biodegradability, film-forming properties, and compatibility with bioactive compounds. By employing techniques such as biopolymer blending, ionic cross-linking, nanoparticle reinforcement, and plasticization, these materials can be engineered into edible films, coatings, and active or intelligent films with enhanced mechanical and barrier performance. These films can also enable controlled release of antimicrobial and antioxidant agents and facilitate real-time freshness monitoring of perishable products such as seafood. Collectively, these advancements underscore the considerable potential of seaweed-based packaging materials to enhance seafood quality and safety, reduce reliance on non-biodegradable plastics, and promote more sustainable food packaging systems. With ongoing technological advancements, seaweed-derived packaging is anticipated to play a key role in advancing sustainable seafood preservation.

## Figures and Tables

**Table 1:** Seaweed-based Packaging Systems and their Application in Seafood Preservation.

Classification *	Seaweed-Derived Material(s)	Other Polymers or Functional Components	Applied Seafood Products	Major Preservation/ Functional Effects	Observed Effects	Ref.
Edible Films & Coatings						
i	Alginate	Gelatin, phycocyanin	Shrimp	Antimicrobial	Slowed bacterial growth; reduced oxidative spoilage; significantly delayed melanosis	[78]
	Carrageenan/Alginate	VCO	Salted Fish	Structural reinforcement/Barrier; Carrier of active compounds	Improved quality (i.e., water content, weight loss, pH, TVB, salt content, appearance & texture) and shelf-life of product	[93]
	Alginate	Glycerol	Fish	Structural reinforcement/Barrier; Carrier of active compounds	Maintained quality of fish during frozen storage	[94]
	Fucoidan	Chitosan, crucian carp protein hydrolysate/nanoliposomes	Fish fillets	Antioxidant; Antimicrobial	Reduced oxidative spoilage and microbial growth; maintained chemical, microbiological, and sensory quality during 16-day refrigerated storage; extended shelf life	[95]
iii	*Gracilaria birdiae* (SP)	Glycerol	Shrimp	Antioxidant; Antimicrobial	Improved chemical and microbial stability; lower psychrotrophic bacterial counts; comparable to glazing for preventing dehydration	[35]
	*Padina tetrastromatica* (extract)	-	Fish fillets	Antioxidant; Antimicrobial	Extended frozen-storage shelf life by 8 weeks; significantly delayed increases in pH, TVB-N, PV, and TBARS.	[75]
Active Packaging						
iii	*Gracilaria crassa* (extract)	Chitosan, AgNPs, Glycerol	Shrimp nuggets	Antioxidant; Antimicrobial	Inhibited bacterial growth (TVC); delayed lipid oxidation (PV, FFA); Extended shelf life	[91]
i/ii	Alginate/Seaweed extracts	-	Frozen fish	Structural reinforcement/Barrier; Antioxidant; Antimicrobial	Reduces freezer burn; reduced film moisture and improved water resistance	[72]
i	Alginate; Fucoidan	Chitosan	Fish fillets	Antioxidant; Antimicrobial; Structural reinforcement/Barrier	Extended shelf life to16 days; reduced TVB-N, TBARS, and TVC; lower weight loss and improved chromaticity; minimal sensory changes.	[70]
ii	Phloroglucinol (or phlorotannin)	Pea starch	Fish	Antioxidant; Antimicrobial	Delayed increase in pH and TMA; reduced microbial growth and oxidation-related volatile compounds; delayed muscle softening and proteolysis; extended refrigerated quality	[81]
Intelligent Packaging						
i	Carrageenan	Arrowroot starch, Kyoho skin extract	Shrimp	Carrier of active compounds; Structural reinforcement/Barrier	Indicator film color shifts (green and yellow) correlated strongly with increases in TVC and pH, reflecting shrimp spoilage levels during storage	[96]
		Konjac glucomannan, blueberry anthocyanin	Shrimp	Carrier of active compounds; Structural reinforcement/Barrier	Improved film properties (tensile strength, hydrophobicity, thermal stability & barrier properties); change color of films (pink to bluish violet) indicating spoilage	[97]
	Alginate	Anthocyanin	Shrimp	Freshness monitoring/Colorimetric indicator	Color change strongly correlated with viable bacterial counts, TVB-N, pH, K-value, H-value, and biogenic amines; differentiated fresh, medium-fresh, and spoiled shrimp	[98]
i/ii	Alginate; Phycoerythrin	Fish gelatin, cellulose nanofibrils	Fish	Structural reinforcement/Barrier; Freshness monitoring/Colorimetric indicator	pH-responsive color change from red through purple–brown to colorless; color change accompanied increases in bacterial counts and TVB-N during storage	[80]
Multilayer & Multi-functional Packaging Systems						
i	Carrageenan	Anthocyanin, cinnamaldehyde	Fish	Carrier of active compounds; Structural reinforcement	Carrageenan protects the active substances and regulates their release; film enhanced physical and antioxidant properties and effectively monitors freshness	[99]
		Anthocyanin (SPA), TiO_2_-doped carbon dots (Ti-CDs)	Shrimp	Carrier of active compounds; Structural reinforcement	Color change (pink to green/yellow) monitors freshness via ammonia sensing; Ti-CDs and SPA provide synergistic antimicrobial and antioxidant activity; maintained quality	[74]
		TiO_2_, anthocyanin, gelatin	Fish (Salmon)	Carrier of active compounds; Structural reinforcement	Films distinct color shift changed color corresponding to fish freshness	[100]
	Agar	Gelatin, anthocyanin, ZnO nanoparticles	Shrimp	Carrier of active compounds; Structural reinforcement	Excellent pH and gas sensitivity for effective freshness monitoring; film with good antioxidant and antibacterial activities, UV light barrier, and high transparency	[101]
	Agar/Carrageenan	Anthocyanin-loaded liposomes	Shrimp	Antioxidant; Antimicrobial; Structural reinforcement	The optimized film showed enhanced mechanical and barrier properties while providing a distinct, reliable color shift from blue to yellow–green during early shrimp spoilage	[102]
	Sodium alginate	Konjac glucomannan, alizarin, cinnamon essential oil pickering emulsion (CSCEO)	Shrimp	Carrier of active compounds; Structural reinforcement	Dual-function system: inner Alg/Al layer provides reversible pH/NH_3_ monitoring (yellow–brown to purplish red); outer Kgm/CSCEO layer acts as a high-barrier bacteriostatic property	[103]
i	Fucoidan	Chitosan, blackcurrant anthocyanin	Fish	Antioxidant; Antimicrobial	Films showed antimicrobial activity and delayed increases in TVB-N and TBA values during storage; anthocyanin showed pH-responsive color changes enabled visual monitoring of tuna freshness	[89]
		Chitosan, *Plectranthus scutellarioides* anthocyanins	Fish	Antioxidant; Antimicrobial	Enhanced antioxidant and antibacterial activity; extended salmon shelf life; Anthocyanin showed visible pH-responsive color changes and ammonia sensitivity enabled monitoring of salmon spoilage	[90]

* Note: Classification includes (i) seaweed polysaccharide-based films and coatings, (ii) packaging systems incorporating isolated seaweed-derived bioactive compounds, (iii) whole seaweed extract-based systems.

**Table 2:** Comparative performance of seaweed-derived, biobased and conventional packaging materials.

Packaging Material	Film Performance	Cost	Biodegradability	Scalability	Key Limitations
Seaweed-based film (e.g., alginate, carrageenan, agar)	Good film formation, oxygen barrier; generally weak moisture resistance and mechanical strength, inherent functional properties [6]	High (depend on processing) [124,125]	High [124]	Moderate [6]	Moisture sensitivity, mechanical weakness, variability of raw material [6]
Starch	Good film formation and oxygen barrier; poor moisture resistance [126]	Low [125]	High [125,126]	High [125]	Hydrophilicity, brittleness, high moisture sensitivity, poor thermal processability [125,126]
Chitosan	Good mechanical properties; Inherent antimicrobial activity; good oxygen barrier [125,126]	Moderate [125,126]	High [126]	Moderate [126]	High sensitivity to water, low mechanical and thermal stability [126]
Cellulose derivatives	Good mechanical strength and transparency; good oxygen barrier [125,126]	Low-Moderate [125,126]	High [126]	Moderate [125]	Moisture sensitivity and chemical modification requirements
PLA	High tensile strength but low flexibility, Good moisture barrier [125]	Low [125,126]	Low [125]	High [125,126]	Brittle, low heat stability [125,126]
PHA	Good mechanical/barrier performance [125]	Moderate [125]	Moderate [125]	Moderate [125,126]	Brittle, narrow thermal processability [127]
Conventional petroleum-based plastics (PE, PP, PET)	Excellent overall mechanical and barrier performance [125,128]	Low [125,126,128]	Low [125,128]	Very high [128]	Fossil-resource dependence, persistence, environmental burden; Without inherent functional/active properties [125,128]

Note: Performance, cost, biodegradability, processing, and scalability are comparative assessments based on reported literature and may vary depending on polymer grade, formulation, processing method, application, and production scale.

## Data Availability

No new data were created or analyzed in this study. Data sharing is not applicable to this article.
